# A Nonclinical Safety Evaluation of Cold Atmospheric Plasma for Medical Applications: The Role of Genotoxicity and Mutagenicity Studies

**DOI:** 10.3390/life14060759

**Published:** 2024-06-13

**Authors:** Piimwara Yarangsee, Supakit Khacha-ananda, Pornsiri Pitchakarn, Unchisa Intayoung, Sirikhwan Sriuan, Jirarat Karinchai, Apiwat Wijaikhum, Dheerawan Boonyawan

**Affiliations:** 1 Department of Forensic Medicine, Faculty of Medicine, Chiang Mai University, Chiang Mai 50200, Thailand; waraluk_ya@cmu.ac.th (P.Y.); ieynoghk@gmail.com (U.I.); sirikhwan_sr@cmu.ac.th (S.S.); 2 Department of Biochemistry, Faculty of Medicine, Chiang Mai University, Chiang Mai 50200, Thailand; pornsiri.p@cmu.ac.th (P.P.); jirarat.ka@cmu.ac.th (J.K.); 3 Research and Innovation Division, Electricity Generating Authority of Thailand, Nonthaburi 11130, Thailand; apiwat.wijaikhum@egat.co.th; 4 Plasma and Beam Physics Research Facility, Faculty of Science, Chiang Mai University, Chiang Mai 50200, Thailand; dheerawan.b@cmu.ac.th

**Keywords:** mutagenicity, genotoxicity, cold air plasma jet, fibroblast cells

## Abstract

Atmospheric nonthermal plasma (ANTP) has rapidly evolved as an innovative tool in biomedicine with various applications, especially in treating skin diseases. In particular, the formation of reactive oxygen species (ROS) and nitrogen species (RNS), which are generated by ANTP, plays an important role in the biological signaling pathways of human cells. Unfortunately, excessive amounts of these reactive species significantly result in cellular damage and cell death induction. To ensure the safe application of ANTP, preclinical in vitro studies must be conducted before proceeding to in vivo or clinical trials involving humans. Our study aimed to investigate adverse effects on genetic substances in murine fibroblast cells exposed to ANTP. Cell viability and proliferation were markedly reduced after exposing the cells with plasma. Both extracellular and intracellular reactive species, especially RNS, were significantly increased upon plasma exposure in the culture medium and the cells. Notably, significant DNA damage in the cells was observed in the cells exposed to plasma. However, plasma was not classified as a mutagen in the Ames test. This suggested that plasma led to the generation of both extracellular and intracellular reactive species, particularly nitrogen species, which affect cell proliferation and are also known to induce genetic damage in fibroblast cells. These results highlight the genotoxic and mutagenic effects of ANTP, emphasizing the need for the cautious selection of plasma intensity in specific applications to avoid adverse side effects resulting from reactive species production.

## 1. Introduction

Atmospheric nonthermal plasma (ANTP), an innovative approach, has shown promising medical properties, such as anti-microorganism and anti-inflammation activity as well as the ability to induce cancer cell apoptosis and accelerated wound healing [1,2,3]. ANTP contains UV photons, neutral or excited atoms and molecules, negative and positive ions, free radicals, and free electrons resulting from the ionization of carrier gas under atmospheric pressure [4]. Reactive oxygen species (ROS) and nitrogen species (RNS) are considered key substances among the free radicals, which accumulate during the plasma–cell interaction and influence cellular responses upon exposure to ANTP [5,6]. The other constituents of plasma such as UV, as well as a mixture of photons, neutral or excited atoms and molecules, negative and positive ions, and free electrons, might affect the cells and generate an effect on DNA. The type of carrier gas used affects the amount and type of reactive substances released from ANTP; helium or argon plasma generates abundant ROS, while nitrogen or a combination of argon and helium plasma produces high levels of RNS [7]. Additionally, a therapeutic effect was observed with a cold air plasma jet (CAPJ) generated from combined helium and argon gas, which exhibited higher activity in inducing wound closure in keratinocyte cells compared to a CAPJ generated from helium gas alone [8]. 

Previous studies have primarily focused on the therapeutic effects of ANTP, revealing significant findings. ANTP has been shown to increase the expression of matrix metalloproteinase-9 and urokinase-type plasminogen activator, while decreasing E-cadherin expression, thus inducing epithelial-to-mesenchymal transition and accelerating wound healing in fibroblast cell lines [9]. Exposure to cold atmospheric argon plasma resulted in the significant release of epidermal growth factor and transforming growth factor-βi, promoting cell proliferation in murine fibroblast cell lines [10]. Clinical studies have demonstrated a highly significant reduction in bacterial load in chronic wound patients treated with cold atmospheric argon plasma [11], as well as a significant improvement in ulcer wound closure after the same treatment [12]. Both ROS and RNS, generated by ANTP, play crucial roles in these biological outcomes. Additionally, the electric field generated during plasma ionization has been reported to influence cell membranes through electroporation, creating pores that allow extracellular molecules (ROS and RNS) to enter the cells [13,14]. 

ROS and RNS generally exhibit a dual role, where low or moderate concentrations have positive effects on cellular function through signaling cascades, while high levels resulting from prolonged exposure lead to oxidative stress and damage to cellular structures [15]. Diseases such as cancer, arthritis, aging, autoimmune disorders, and cardiovascular and neurodegenerative diseases have been associated with oxidative stress [16], and free radicals have been linked to genetic material damage, including DNA mutation and cancer development [17]. Although ANTP has shown efficacy and positive clinical outcomes for medical treatment, ensuring patient safety is crucial when applying it in clinical settings to avoid side effects on genetic material. Therefore, the careful selection of plasma devices for clinical practice is essential to minimize harm to healthy cells, and appropriate plasma dosages must be closely controlled depending on the treatment type [18]. However, the lack of universally accepted guidelines for plasma dosage, various plasma device types, and differing treatment conditions make it challenging to compare the biological effects and determine safe plasma applications [19]. In this study, we present our team’s newly developed air plasma jet with the goal of applying it in clinical medicine, particularly for wound treatment in chronic diabetes patients and other types of wounds. We utilized a newly designed CAPJ that uses atmospheric air as a carrier gas, making it highly portable and suitable for clinical units in hospitals without requiring nitrogen, helium, or argon gas. In addition, the advantage of using air as a carrier gas is that it reduces the temperature when applied to human skin. The previous report related to plasma generation was based on argon or helium gas as a carrier gas to generate plasma; however, our study provided safety information of plasma devices which use air as a carrier gas. Previous studies by Thana in 2019 and 2020 [20,21] demonstrated the generation of reactive oxygen and nitrogen species (RONS) such as nitric oxide, hydroxyl radical, atomic oxygen, and ozone using a compact low-temperature plasma jet device similar to a CAPJ, with a generated plasma temperature of approximately 35 °C. These studies also observed antibacterial activity and noncytotoxicity to dermal fibroblasts. Despite the benefits of plasma technology to various medical fields, the authors point out that different cold atmospheric plasma devices generate varying plasma compositions and treatment times, necessitating assessments of efficacy and safety. Therefore, it is essential to conduct safety studies, especially for the genotoxicity and mutagenicity of ANTP, to avoid severe side effects. This study aims to evaluate the effect of ANTP, specifically a CAPJ named Nightingale, on adverse genetic responses in NIH3T3 murine fibroblasts, with the objective of understanding the cellular response mechanisms after ANTP exposure.

## 2. Materials and Methods

### 2.1. Atmospheric Nonthermal Plasma (ANTP) Instrument

**Figure 1:**
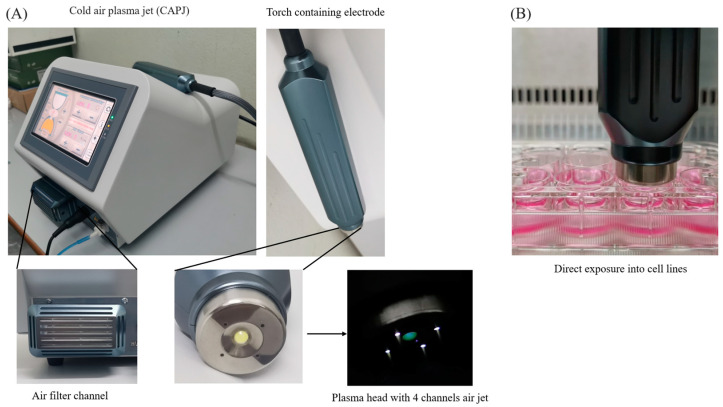
The Nightingale instrument. (**A**) The instrument consisted of an air filter channel to pump atmospheric air into the plasma head containing an electrode, in which plasma was generated under a high electromagnetic field and released through the plasma head via four channels of air jets. (**B**) The direct exposure of plasma to the NIH3T3 murine fibroblasts which were cultured in DMEM medium at a distance of approximately 1.6 cm from the surface of the cell lines.

The CAPJ (Figure 1A) in a model called “Nightingale” has been certified as a standard class 2a medical device according to the Food and Drug Administration with the Conformité Européene (CE) mark, which covers electrical medical equipment (IEC60601-1 and IEC60601-2), and software validation IEC62304. It produced air plasma in transient mode with a plasma temperature below 40 °C. The instrument was set up in a control room with ambient temperature at 27.5 ± 0.95 °C and relative humidity of 61.0 ± 6.0%. The ambient air was used as a carrier gas for plasma generation with different flow rates of 3, 5, 7, 9, and 11 L/min. The plasma is produced by a burst repetition rate of 2 kHz, and it was released from the plasma head via a four-channel air jet with 4, 7, and 10 pulse groups per second (plasma intensity) [21]. During the experiment, the NIH3T3 murine fibroblasts, which were cultured in a culture medium, were directly exposed to plasma that was generated through the plasma head, as shown in Figure 1B.

### 2.2. Cell Culture

The murine fibroblast cell lines (3T3-L1; ATCC no. ATCC^®^ CL-173™) were cultured in Dulbecco’s modified eagle medium (DMEM) supplemented with 10% heat-inactivated fetal bovine serum (Hyclone, Logan, UT, USA), penicillin (100 units/mL), and streptomycin (100 µg/mL) in a humidified atmosphere of 5% CO_2_ at 37 °C. The cells were harvested using 0.05% trypsin–EDTA solution and washed twice in phosphate-buffered saline (PBS). Then, the cells were centrifuged and resuspended with DMEM. The cells were stained with trypan blue solution and counted under a light microscope for cell viability evaluation. The number of cells was adjusted to the desired cell number.

### 2.3. Exposure of Plasma to the Murine Fibroblast Cells 

The NIH3T3 murine fibroblasts were directly exposed to plasma generated by Nightingale for 30 s in all experiments. The experiment for this study was grouped according to plasma intensity exposure (0, 4, 7, and 10 pulses) coupled with different air flow rates (3, 5, 7, 9, and 11 L/min). The murine fibroblast cells which were exposed to a different airflow without plasma generation were classified as a plasma intensity of 0 pulses. After cytotoxic and cell proliferation testing, the plasma intensity at IC_30_ (30% inhibitory concentration) was chosen for further analysis according to ISO10993-5. This guide recommends that treatment should be classified as a substance affecting cell viability and proliferation if it results in a decrease of 30% or more [22,23].

### 2.4. Cell Viability Testing

To investigate the dose toxicity of plasma on the viability of murine fibroblast cells, the cell viability was determined by a 3-(4,5-dimethylthiazol-2-yl)-2,5-diphenyl tetrazolium bromide (MTT) assay [24]. The MTT assay is used to measure cellular metabolic activity as an indicator of cell viability, proliferation, and cytotoxicity. The murine fibroblast cells (2 × 10^5^ cells) were cultured on sterile 12-well tissue culture plates in a humidified atmosphere of 5% CO_2_ at 37 °C. The cells were exposed to plasma for 30 s and further incubated for 24 h. After that, the cytotoxicity was measured by adding 2 mg/mL of MTT reagent into the wells. The blue formazan crystal was then dissolved in dimethyl sulfoxide (DMSO). The absorbance at wavelengths of 540 and 630 nm was measured by a microplate reader (BioTek, Winooski, VT, USA). The percentage of cell viability was calculated by comparing it with the negative control (cells treated with a medium). 

### 2.5. Colony Formation Assay

To investigate the adhesion-independent cell proliferation after exposure of the murine fibroblast cells to plasma, the colony formation assay modified from previous publications was performed [24,25]. The cells (2 × 10^5^ cells) were cultured on sterile 12-well tissue culture plates in a humidified atmosphere of 5% CO_2_ at 37 °C. The cells were exposed to plasma for 30 s, and then, the plate was further incubated for 24 h. After that, the cells were harvested and stained with trypan blue solution for cell viability testing. Five-hundred viable cells were placed on sterile 6-well tissue culture plates, and the plates were incubated at 37 °C in a humidified atmosphere of 95% containing 5% CO_2_ for seven days. Finally, the colonies were stained with 0.5% *w*/*v* of crystal violet for 20 min. The number of colonies consisting of at least 50 cells was counted under an inverted microscope (Olympus, Tokyo, Japan). The percentage of colony formation was calculated by comparing with the negative control (cells treated with a medium). 

### 2.6. Investigation of Intracellular Reactive Oxygen (ROS) and Nitrogen Species (RNS)

To investigate the generation of ROS and RNS in the plasma-exposed cells, the percentage of the treated cells which produced intracellular ROS and RNS was determined by a flow cytometer. The cells (2 × 10^5^ cells) were cultured on sterile 12-well tissue culture plates in a humidified atmosphere of 5% CO_2_ at 37 °C. The cells were then exposed to plasma for 30 s, and the plate was further incubated for 24 h. After incubation, the plasma-exposed cells were harvested and washed twice with sterile phosphate-buffered saline (PBS). The intracellular ROS were analyzed by staining the cells with 2′,7′-dichlorofluorescin diacetate (DCFDA) according to the manufacturer’s instructions (Sigma-Aldrich, St. Louis, MO, USA) and 2 mM hydrogen peroxide (H_2_O_2_) was used as a positive control. The intracellular RNS were also measured by staining the cells with 4-amino-5-methylamino-2′,7′-difluorofluorescein diacetate (DAF-FM Diacetate) according to the manufacturer’s instructions ((Sigma-Aldrich, St. Louis, MO, USA), and 1 mM diethylamine NONOate sodium salt was used as a positive control. The fluorescent-stained cells were counted using a flow cytometer. The result was expressed as the percentage of fluorescent-stained cells.

### 2.7. Quantification of Extracellular ROS and RNS

To quantify the ROS mediator in the culture medium after exposing the medium to plasma, the plasma-exposed culture medium was collected to measure hydrogen peroxide (H_2_O_2_) by a hydrogen peroxide assay kit according to the manufacturer’s instructions (Cayman Chemical, Ann Arbor, MI, USA). The plasma-exposed culture medium was harvested and mixed with an enzyme reaction solution which consisted of hydrogen peroxide detector ADHP and horseradish peroxidase in a separate 96-well plate. After that, the fluorescence intensity was immediately measured at a wavelength of 530 nm excitation and 585 nm emission by a microplate reader (BioTek, Winooski, VT, USA). The H_2_O_2_ level was calculated from the standard curve. As a quantification of the RNS mediator in the culture medium, the plasma-exposed culture medium was collected to measure nitric oxide (NO) by the Griess reagent system [26]. The plasma-exposed culture medium was mixed with Griess reagent containing 0.1% naphthylethylenediamine dihydrochloride and 1% sulfanilamide in 5% phosphoric acid in water in a separate 96-well plate. The plate was incubated for 15 min. The absorbance of the reaction was measured at a wavelength of 550 nm by a microplate reader (BioTek, Winooski, VT, USA). The nitric oxide level was calculated from the sodium nitrite (NaNO_2_) standard curve [27]

### 2.8. Mutagenicity Testing

#### 2.8.1. 8-Hydroxy-2′-deoxyguanosine (8-OHdG) Quantification

To quantify the by-product of DNA adduct from reactive species in the plasma-exposed cells, the quantification of 8-OHdG was performed according to the manufacturer’s guidelines. The cells (2 × 10^5^ cells) were cultured on sterile 12-well tissue culture plates in a humidified atmosphere of 5% CO_2_ at 37 °C. The cells were exposed to plasma for 30 s and further incubated for 24 h; the plasma-exposed cells were harvested and washed twice with sterile phosphate-buffered saline (PBS). The 8-OHdG ELISA Kit (Elabscience, Hubei, China) was used for 8-OHdG analysis according to the manufacturer’s instructions. The absorbance of the reaction was measured at a wavelength of 540 nm by a microplate reader (BioTek, Winooski, VT, USA). The positive control for 8-OHdG was the cells treated with 2 mM of H_2_O_2_. The level of 8-OHdG was calculated from the standard curve. 

#### 2.8.2. DNA Strand Break Analysis

To investigate DNA strand breaks in plasma-exposed cells, the TUNEL assay was performed according to the manufacturer’s guidelines. The cells (2 × 10^5^ cells) were cultured on sterile 12-well tissue culture plates in a humidified atmosphere of 5% CO_2_ at 37 °C. The cells were exposed to plasma for 30 s and further incubated for 24 h; the plasma-exposed cells were harvested and washed twice with sterile phosphate-buffered saline (PBS). The APO-BRDU™ Kit (BD Biosciences, Franklin Lakes, NJ, USA) was used for DNA strand break analysis according to the manufacturer’s instructions. The fluorescent-stained cells were counted using a flow cytometer. The positive control was the cells that were treated with 2 mM H_2_O_2._ The result was expressed as the percentage of fluorescent-stained cells.

To confirm DNA damage, particularly double-strand breaks (DSBs), and to assess the cellular response to genotoxic stress, the detection of gamma H2AX (γ-H2AX) was performed by immunofluorescent staining with a slightly modified method [28]. After incubating the cells with plasma, the plasma-exposed cells were harvested and washed twice with sterile phosphate-buffered saline (PBS). The cells were fixed with 4% paraformaldehyde (Sigma-Aldrich, St. Louis, MO, USA) and permeabilized with 0.1% Triton X-100. The nonspecific binding was blocked by adding blocking solution. The cells were stained with anti-human γ-H2AX antibody (Merck, Darmstadt, Germany) for 1 h at room temperature followed by staining with anti-rabbit IgG conjugate FITC (Dako, Glostrup, Denmark) for 1 h at room temperature. Finally, the cells were counterstained with 1 µg/mL of 4′,6-diamidino-2-phenylindole (DAPI) solution (PanReac AppliChem, Barcelona, Spain). The fluorescent-stained cells were observed under an inverted microscope, version Axio Observer 7 (Carl Zeiss, Aalen, Germany). 

#### 2.8.3. Bacterial Reverse Mutation Assay

The determination of the mutagenicity of plasma was performed by the Ames test according to the protocol of OECD test no.471 [29]. However, the preparation of test substances in our study could not follow this guideline since they were not solid or liquid test substances. Hence, the protocol for plasma exposure was modified from a previous study by Patenall et al., 2021 [30]. Additionally, Mortelmansit and Zeiger, 2000 [31], recommended that for general screening purposes, a tier approach should be used using strains TA98 and TA100 with or without metabolic activation to ensure that mutagens with different mechanisms of action can be detected. Prior to the mutagenicity testing, the effects of plasma on bacterial viability and growth curve were observed. Two hundred microliters of diluted bacteria was added to sterile 96-well tissue culture plates. The optical density of bacteria was measured at a wavelength of 600 nm every 10 min for 24 h to represent the growth rate. Two strains of *Salmonella typhimurium* (TA98 and TA100) were exponentially grown in Oxoid nutrient broth at a temperature of 37 °C. For the Ames test, specifically, we used the pre-incubation method. One hundred microliters of bacteria suspended in phosphate buffer was added into sterile 12-well tissue culture plates and directly exposed to plasma at various air flow rates and plasma intensities for 30 s. After plasma treatment, the culture was left to stand for 30 min before being mixed with a surface agar solution containing histidine and biotin. For 2-(2-furyl)-3-(5-nitro-2-furyl)-acrylamide (AF-2) (Wako Pure Chemicals, Osaka, Japan) as a positive control, 100 μL of bacterial suspension was mixed with buffer and AF-2 (0.1 ug/plate for TA98 and 0.01 ug/plate for TA100). The plasma-exposed bacteria were mixed with a surface agar solution consisting of histidine and biotin prior to plating on bottom agar. The culture plates were continuously incubated at 37 °C for 48 h. Finally, the number of revertant colonies was counted under a stereomicroscope. The mutagenic index was calculated from the ratio between revertant colonies in the treatment group and control group. The mutagenicity in CAPJ-exposed bacteria was compared to the unexposed bacteria. 

### 2.9. Statistical Analysis

Experimental data are expressed as the mean and standard deviation of three independent experiments for each test. GraphPad Prism Version 9 was used to evaluate the statistical significance between experiment groups. One-way analysis of variance (ANOVA) and an independent sample t-test were performed for paired comparisons between the unexposed and the CAPJ-exposed cells. Statistically significant differences are expressed with *p* < 0.05.

## 3. Results

### 3.1. Fibroblast Cell Viability

**Figure 2:**
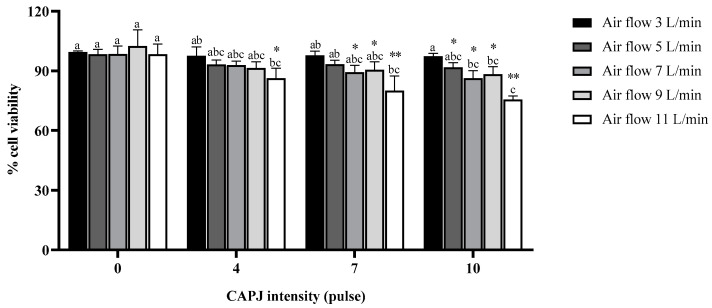
The cell viability of fibroblast cells after exposure with plasma. The cells were exposed to different air flow rates coupled with plasma. The percentage of fibroblast cell viability is expressed as mean and standard deviation. * and ** indicate a statistical difference in cell viability, comparing plasma intensities of 4, 7, and 10 pulses to a plasma intensity of 0 pulses at the same air flow rate (* *p* value < 0.05; ** *p* value < 0.01). The lower-case letters indicate a statistical difference in cell viability comparing the same plasma intensity. Data are representative of three independent experiments.

To demonstrate the effect of plasma on murine fibroblast viability, the cells were exposed to plasma for 30 s and further incubated for 24 h. The MTT assay was used for evaluating cell viability. The percentage of cell viability in the treatment groups was calculated by comparing with the negative control. No significant difference in cell viability was observed at 0 pulse intensity coupled with different air flow rates, indicating that the air released from the plasma head did not affect cell viability. There was also no significant difference in cell viability among the cells exposed to all intensities of plasma at a flow rate of 3 L/min. However, cell viability tended to decrease in a dose-dependent manner after exposure to plasma at different air flow rates (5, 7, 9, and 10 L/min) and intensities (4, 7, and 10 pulses), as shown in Figure 2. Notably, the cell viability was significantly reduced when exposed to plasma with all intensities (4, 7, and 10 pulses) coupled with an air flow rate of 11 L/min compared to treatment with 0 pulse intensity under the same air flow rate. Interestingly, the highest toxicity was observed in cells exposed to a plasma intensity of 10 pulses under an air flow rate of 11 L/min.

### 3.2. Suppression of Cell Proliferation 

**Figure 3:**
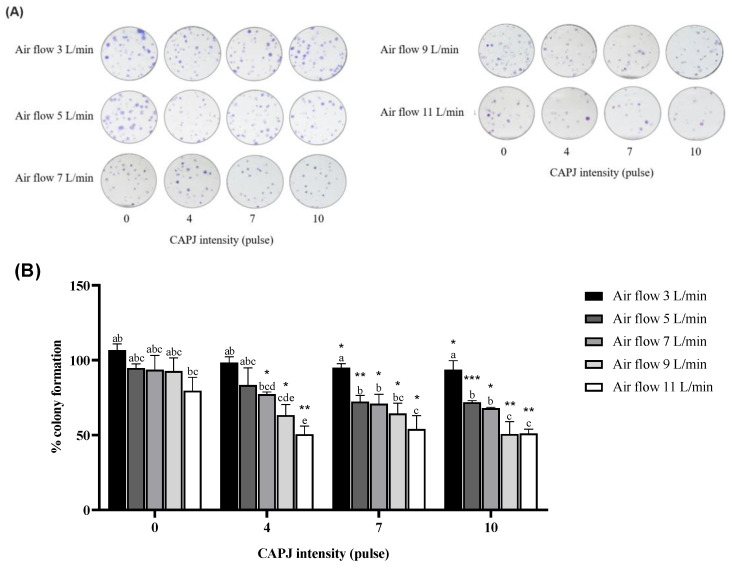
The suppression of cell proliferation after exposure to plasma. (**A**) The proliferative effect of plasma on the plasma-exposed cells demonstrated by a colony formation assay. The surviving colonies were stained and counted. (**B**) The percentage of colony formation is expressed as mean and standard deviation. *, **, and *** indicate a statistical difference in the percentage of colony formation, comparing plasma intensities of 4, 7, and 10 pulses to a plasma intensity of 0 pulses at the same flow rate (* *p* value < 0.05; ** *p* value < 0.01; *** *p* value < 0.001). The lower-case letters indicate a statistical difference in cell viability comparing the same plasma intensity. Data are representative of three independent experiments.

To assess the effect of plasma on fibroblast cell proliferation, the colonies of plasma-exposed cells were formed over seven days and stained with crystal violet. The percentage of colony formation in the treatment groups was calculated by comparing it with the negative control. The results demonstrated a tendency for the ability to form colonies to decrease with increasing air flow rates when directly exposed to plasma at all intensities (4, 7, and 10 pulses), as shown in Figure 3A. At a plasma intensity of 0 pulses, the colony formation in the cells exposed to plasma coupled with an air flow rate of 11 L/min appeared to decrease compared to other flow rates. Furthermore, under the same plasma intensity treatment, our findings indicated that the percentage of cell proliferation significantly decreased in response to increasing air flow rates. Comparing the same air flow rate, we observed a significant reduction in colony formation for plasma-exposed cells at intensities of 4, 7, and 10 pulses compared to a plasma intensity of 0 pulses. The plasma-exposed cells at intensities of 7 and 10 pulses coupled with all air flow rates demonstrated significantly fewer forming units (Figure 3B). In addition, the colony formation was performed after exposure of the cells to plasma after 24 h of incubation; the reduction in colony formation only represents cell death that occurred more than 24 h after exposure.

### 3.3. Production of Intracellular Reactive Oxygen (ROS) and Nitrogen Species (RNS) 

**Figure 4:**
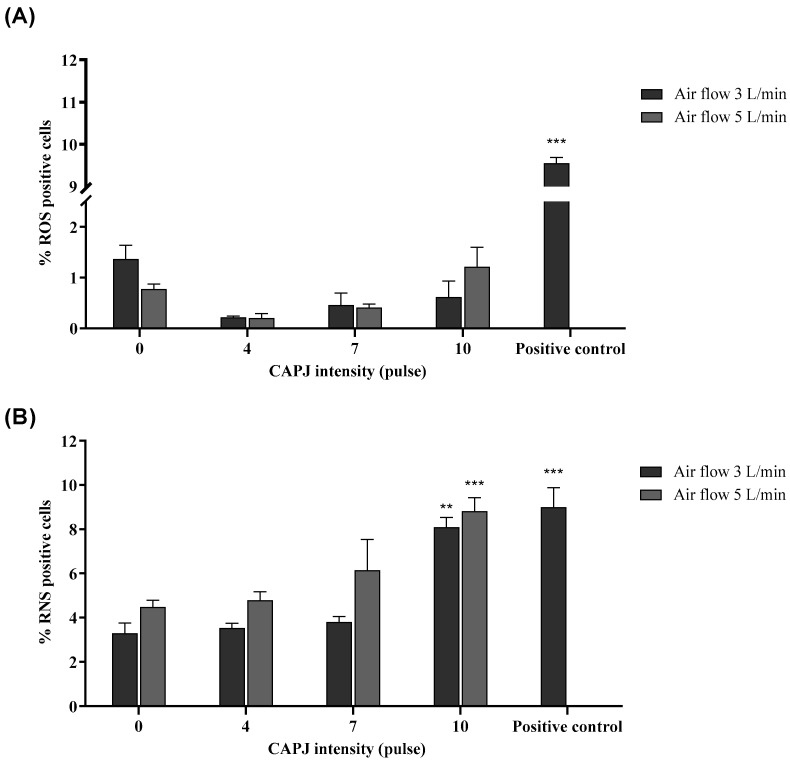
Modulation of intracellular ROS and RNS. (**A**) Percentage of ROS-positive cells in plasma-exposed cells. The treated cells were harvested to stain them with 2′,7′-dichlorofluorescin diacetate. The stained cells were counted by a flow cytometer. (**B**) Percentage of RNS-positive cells in plasma-exposed cells. The treated cells were harvested to stain them with 4-amino-5-methylamino-2′,7′-difluorofluorescein diacetate. The stained cells were counted by a flow cytometer. Hydrogen peroxide and diethylamine NONOate sodium salt were used as positive controls for ROS and RNS, respectively. The percentage of ROS- and RNS-positive cells was expressed as mean and standard deviation. ** and *** indicate a statistical difference, comparing plasma intensities of 4, 7, and 10 pulses as well as the positive control to a plasma intensity of 0 pulses (** *p* value < 0.01; *** *p* value < 0.001). Data are representative of three independent experiments.

In a previous experiment, it was demonstrated that some intensities of plasma showed a deleterious effect on cell viability and proliferation. However, the toxicological mechanism of plasma on the murine fibroblasts was unclear. Plasma intensity at about IC_30_ (30% inhibitory concentration) was chosen for further experiments. The percentage of ROS-stained cells in the negative control was 1.26 ± 0.12%, which was not statistically significant compared to the cells exposed to a plasma intensity of 0 pulses. The percentage of ROS-stained cells in the positive control (H_2_O_2_) was 9.55 ± 0.14%. We found no statistical difference between the percentages of ROS in the cells which were exposed to plasma at intensities of 0, 4, 7, and 10 as shown in Figure 4A. As for RNS quantification, the percentage of RNS-stained cells in the negative control was 2.24 ± 0.11%. The percentage of RNS-stained cells in the positive control (diethylamine NONOate sodium salt) was 8.99 ± 2.55%. The treatment of the cells with plasma at an intensity of 10 coupled with air flow rates of 3 and 5 L/min significantly increased RNS accumulation by approximately two-fold compared to a plasma intensity of 0 pulses at the same flow rate. Furthermore, we also compared the generation of intracellular RNS by exposure to a plasma intensity of 10 pulses with different flow rates (3 and 5 L/min); however, there were no significant differences in the percentage of RNS-positive cells between the two air flow rates (Figure 4B).

### 3.4. Generation of Extracellular Reactive Oxygen (ROS) and Nitrogen Species (RNS) 

**Figure 5:**
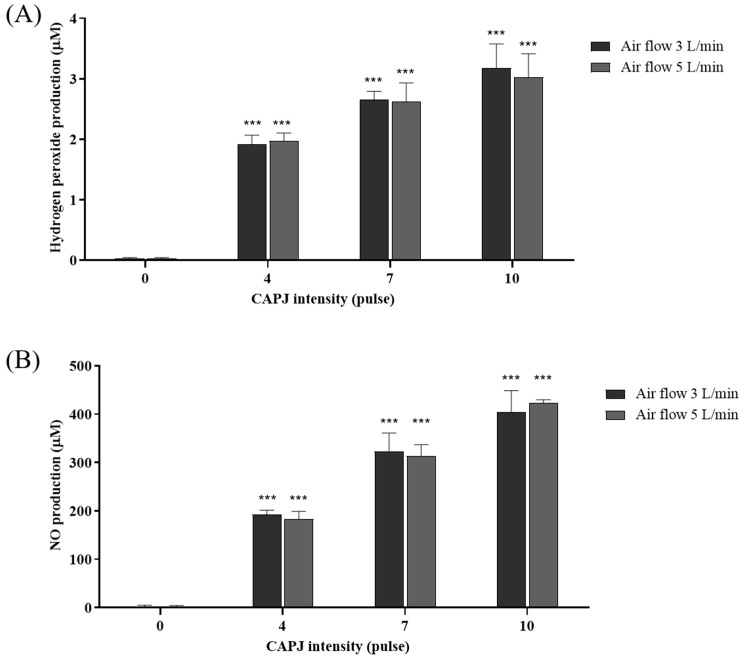
The quantification of extracellular (**A**) H_2_O_2_ and (**B**) NO in the plasma-exposed culture medium. The culture medium was directly exposed to plasma for 30 s; then, the culture medium was harvested and reacted with the specific reagent. The amount of H_2_O_2_ and NO was calculated from the standard curve and expressed as mean and standard deviation. *** indicates a statistical difference, comparing plasma intensities of 4, 7, and 10 pulses to a plasma intensity of 0 pulses at the same flow rate (*** *p* value < 0.001). Data are representative of three independent experiments.

As indicated by the data from the previous experiment, there was a clear increase in reactive species levels in the plasma-exposed cells. We hypothesized that plasma releases reactive species into the environment or other reactive species, leading to an effect on the cellular response. Consequently, these reactive substances could easily diffuse across cellular membranes or induce oxidative stress in the cells. The plasma-exposed medium showed the presence of extracellular ROS and RNS, represented by H_2_O_2_ and NO, respectively (Figure 5). There was a significant difference in the amount of extracellular H_2_O_2_ and NO in the plasma-exposed medium across all plasma intensities. The exposure of the culture medium to plasma generated from Nightingale at intensities of 4, 7, and 10 pulses increased the H_2_O_2_ and NO concentrations by approximately 100-, 160-, and 200-fold, respectively, compared to plasma at an intensity of 0 pulses.

### 3.5. Induction of DNA Damage

**Figure 6:**
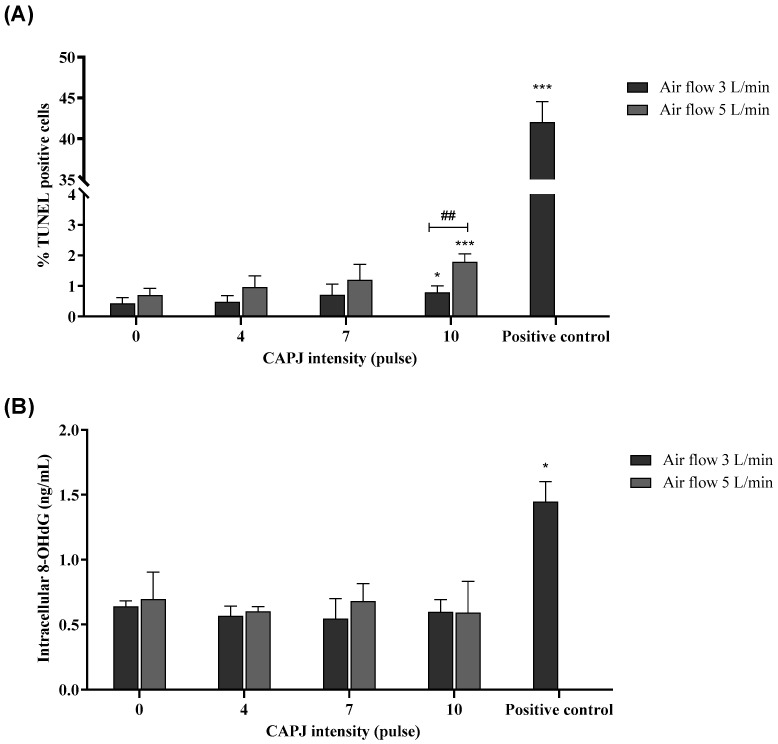
The DNA damage was measured by (**A**) the percentage of TUNEL-positive cells and (**B**) the amount of intracellular 8-OHdG in plasma-exposed cells. The TUNEL assay was used for investigating DNA strand break. The commercial ELISA kit was also chosen for the quantification of 8-OHdG. H_2_O_2_ was used as a positive control. The data were expressed as mean and standard deviation. * and *** indicate a statistical difference, comparing plasma intensities of 4, 7, and 10 pulses as well as the positive control to a plasma intensity of 0 pulses (* *p* value < 0.05 and *** *p* value < 0.001). ^##^ indicates a statistical difference, comparing air flow rates of 3 and 5 L/min (^##^ *p* value < 0.01). Data are representative of three independent experiments.

**Figure 7:**
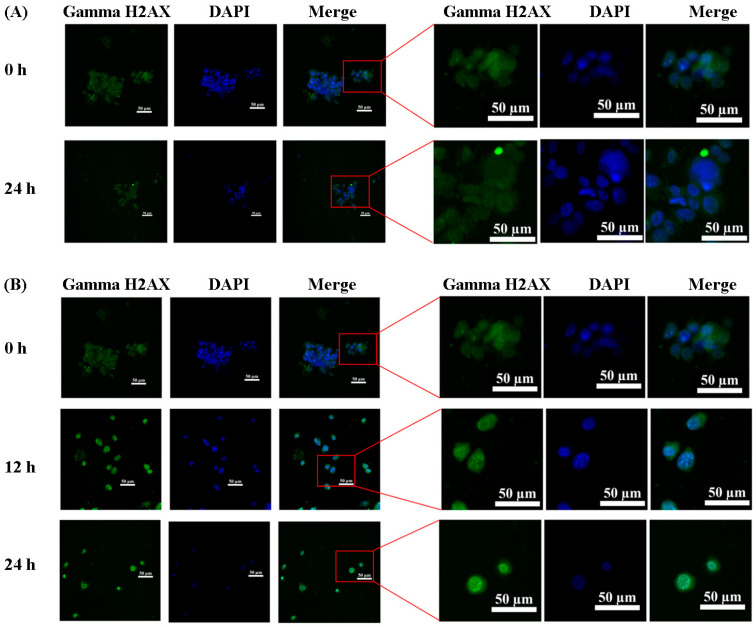
The immunofluorescent staining of gamma H2AX (γH2AX) on plasma-exposed cells. (**A**) Control cells were stained with gamma H2AX at 0 and 24 h. The nucleus was counterstained with DAPI. The fluorescent-staining cells were observed and photographed under an inverted microscope. (**B**) Plasma-exposed cells were exposed to a CAPJ at an intensity of 10 pulses and under an air flow rate of 5 L/min for 30 s, then further incubated for 12 and 24 h. The cells were stained with gamma H2AX (γH2AX) and counterstained with DAPI. Green dot shows γ-H2AX foci, blue nuclei stained with DAPI.

Due to the significant stimulation of reactive species in the culture medium and the murine fibroblast cells observed after plasma exposure in the previous experiment, and considering that reactive species have been reported to attack genetic material such as DNA, leading to DNA damage, we performed a TUNEL assay to investigate the effect of ROS and RNS on the genetic material in the plasma-exposed cells. The percentage of TUNEL-positive cells in the negative control was 0.50 ± 0.30%, which was not statistically significant compared to the cells exposed to a plasma intensity of 0 pulses. The percentage of TUNEL-positive cells in the positive control (2 mM of H_2_O_2_) was 42.02 ± 2.52%. The TUNEL assay revealed an increasing percentage of TUNEL-positive cells, indicating DNA damage. Specifically, at an intensity of 10 pulses and under air flow rates of 3 and 5 L/min, the percentage of TUNEL-positive cells significantly increased compared to the intensity 0 pulses. Notably, the plasma intensity of 10 pulses under an air flow rate of 5 L/min showed a significantly higher percentage of DNA damage compared to the same intensity under an air flow rate of 3 L/min (Figure 6A). Moreover, the detection of γH2AX focus formation was performed to confirm DNA double-strand breaking at an intensity of 10 pulses and under an air flow rate of 5 L/min. The result showed that plasma-exposed cells had the positive staining of γ-H2AX foci at 24 h after exposure to plasma (Figure 7). Furthermore, we measured the by-products of oxidatively damaged DNA formed by ROS and RNS, specifically 8-OHdG. The levels of 8-OHdG in the negative control and positive control (2 mM of H_2_O_2_) were 0.46 ± 0.12 and 1.44 ± 0.16 ng/mL, respectively. The results, shown in Figure 6B, demonstrate no significant difference in the amount of 8-OHdG among the cells exposed to different plasma intensities.

### 3.6. Mutagenicity Potential of Plasma

**Figure 8:**
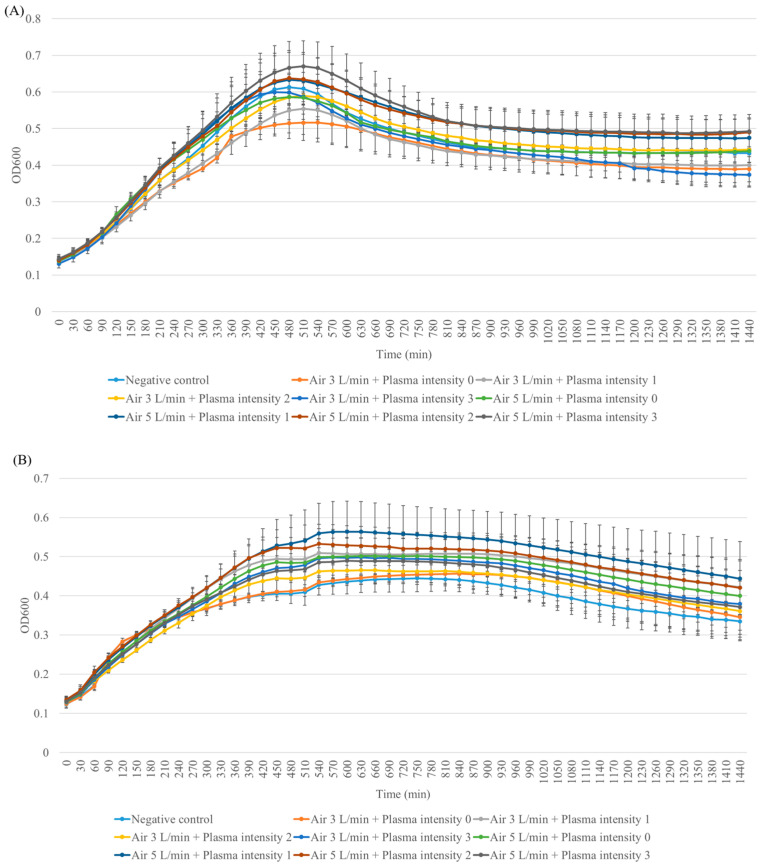
The growth rate of plasma-exposed bacteria was assessed to test the effect of plasma on the bacterial growth of (**A**) strain TA98 and (**B**) strain TA100. After exposure to two strains of bacteria with plasma, the plasma-exposed bacteria were diluted and added to a sterile 96-well plate. The plate was then incubated at 37 °C for 24 h. The optical density (OD) at a wavelength of 600 nm was measured every 10 min. The growth curve is represented with the *x*-axis indicating time of incubation and the *y*-axis indicating OD600 nm. The results are expressed as the average and standard deviation of triplicate experiments.

**Figure 9:**
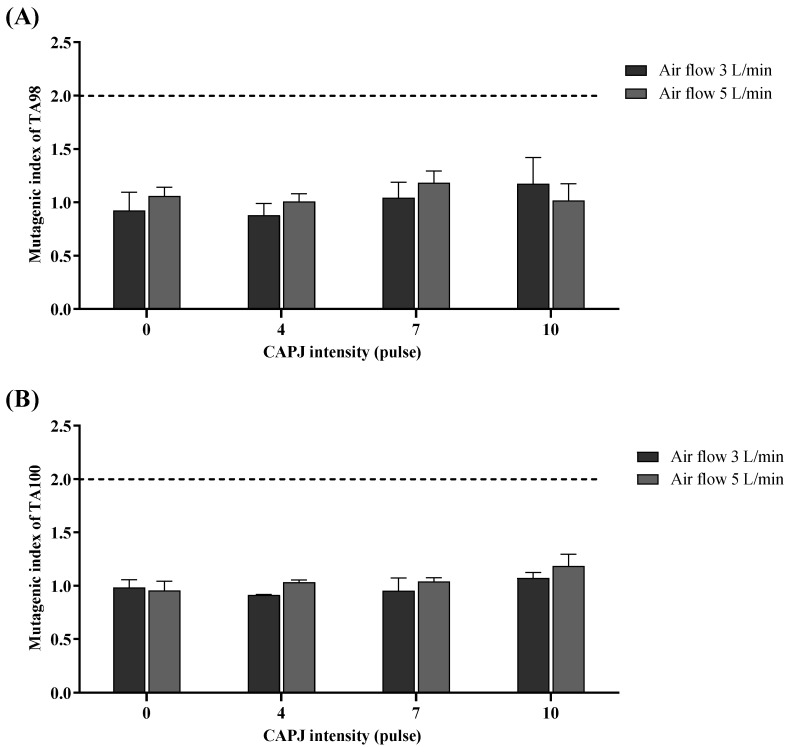
The mutagenicity of plasma was tested on (**A**) *Salmonella typhimurium* strain TA98 and (**B**) *S. typhimurium* strain TA100. The bacteria were directly exposed to plasma for 30 s. After that, the treated bacteria were cultured on a culture medium. The number of revertant colonies was counted. The mutagenic index was calculated and is expressed as mean and standard deviation. The mutagenicity of the test substance was classified by a mutagenic index of more than 2 (over the dot line). Data are representative of three independent experiments. The dotted line represents the cut-off to indicate the test substance is not classified as mutagens (mutagenic index less than 2).

In light of the plasma-induced DNA strand breaks observed in previous experiments, the mutagenic potential of plasma was assessed in the current study using the *Salmonella typhimurium*/reverse mutation assay. Two strains of bacteria (TA98 and TA100) were exposed to different plasma intensities. To evaluate the effect of plasma on bacterial viability, the result showed that the viability of both TA98 and TA100 plasma-exposed bacteria did not differ from the negative control or air control (air flow rate without plasma). Likewise, the growth rate of both TA98 and TA100 bacteria after exposure to plasma did not differ from the growth curve of the negative control or air control (air flow rate without plasma) (Figure 8). From these results, it was confirmed that the plasma did not interfere with the viability and growth rate of *S. typhimurium* (TA98 and TA100). For Ames’s test, the number of revertant colonies in the negative control and plasma exposure did not show significant difference. This suggests that plasma did not exhibit inhibitory effects on bacterial growth. Also, the number of revertant colonies of TA98 exposed to plasma at intensities of 4, 5, and 10 pulses did not show a significant increase compared to plasma-treated bacteria at an intensity of 0 pulses. Although the number of revertant colonies of TA100 exposed to plasma at an intensity of 10 pulses under an air flow rate of 5 L/min significantly increased compared to the number of revertant colonies subjected to a plasma intensity of 0 pulses, according to the mutagenic index, the results indicated that all CAPJ intensities under all air flow rates were not classified as mutagens (mutagenic index less than 2) [32]. Moreover, the mutagenic index in TA100 exposed to plasma at an intensity of 10 pulses under an air flow rate of 5 L/min significantly increased compared to the number of revertant colonies treated with a plasma intensity of 0 pulses (Table 1 and Figure 9). 

## 4. Discussion

Atmospheric nonthermal plasma (ANTP) has found extensive applications in dermatology due to its composition rich in reactive oxygen (ROS) and nitrogen species (RNS), which exhibit antimicrobial, anti-inflammatory, cancer cell death induction, and wound healing acceleration properties [3]. However, inappropriate levels of reactive species without proper host defense mechanisms can lead to adverse side effects in target cells. Reactive species can damage intracellular targets such as proteins, lipids, carbohydrates, and genetic substances, thereby triggering genotoxicity and mutagenicity, which are significant factors in cancer development [33,34]. Thus, determining the genotoxicity of ANTP is crucial for controlling the plasma dose during medical treatment applications. Additionally, the development of medical devices requires the evaluation of biocompatibility, including cytotoxicity and genotoxicity, according to International Standards 10993-3 and 10993-5 (ISO 10993) [23,35]. The air plasma device in our study has been newly developed by our team with the goal of applying it in clinical medicine, especially for wound treatment in chronic diabetes patients and other types of wounds. Therefore, a skin model using NIH3T3 murine fibroblasts was selected. Previous experiments have demonstrated that these cells are suitable target cells for studying the pharmacological and toxicological properties of skin-affected chemical substances. Moreover, they are easy to handle and can be cultured for several passages compared to human primary fibroblast cells [36]. Two methods for ANTP treatment on cells were recommended: direct and indirect exposure. Direct exposure involved treating the cells which were cultured in a culture medium with ANTP, allowing short- and long-lived reactive species to interact with the target. Indirect exposure involved treating the liquid medium with ANTP and transferring it into the cells, resulting in only long-lived species such as H_2_O_2_, NO_2_^–^, and NO_3_^−^. Short-lived species like •OH, ^1^O_2_, •NO, ONOO^–^-) were degraded due to the time delay between treatments [37,38].

The plasma device in our study is based on the production of plasma between two electrodes and requires a carrier gas to transport the active agents onto the skin. The previous report by Brehmer et al. (2014) [39] showed that plasma treatment with the PlasmaDerm^®^ VU-2010 device is safe and effective for patients with chronic venous leg ulcers. However, some side effects such as hyperthermia, redness of the leg, and pain during a single plasma application were observed. It is important to note that the PlasmaDerm^®^ VU-2010 device utilizes a dielectric barrier discharge plasma generator, which uses the skin as the counter electrode, which is different from our device. Moreover, the noteworthy aspect of our study is the utilization of air as a carrier gas in Nightingale, which facilitates practical implementation. Besides using air as a carrier gas, the safety of argon-generated plasma has been studied. Gui min Xu et al. (2015) [40] found that wounds treated with argon-generated plasma showed significantly enhanced improvement compared to the control group. However, overdoses of plasma suppressed wound healing by causing cell death through apoptosis or necrosis. It is assumed that both positive and negative effects may be related to the presence of reactive oxygen and nitrogen species. Several previous studies have been conducted on the kINPen^®^ MED product, the first cold plasma jet using argon. These studies have shown beneficial effects in chronic wound treatment, including wound surface reduction and time to wound closure, independent of background infection [41]. Furthermore, there was no evidence of genotoxicity, but there was induced mortality in chick embryos when kINPen^®^ was applied at a flow rate of 5 L/min [42]. Additionally, there was no observation of mutagenicity in V79 cell lines after exposure to kINPen^®^ at a flow rate of 3 L/min [43]. The difference between plasma generated from argon and air lies in its temperature and composition. Argon plasma exhibits a clear transition from a hot plume to a cold plasma jet. Furthermore, nitrogen species radicals are generated by air plasma, whereas these species produced by nitrogen, oxygen, and carbon dioxide plasmas are below the minimum detection limit [44].

We determined the viability and proliferation of fibroblast cells after exposure to plasma using MTT and colony formation assays, respectively. We observed a significant dose-dependent decrease in cell viability and proliferation after plasma exposure. Similar reductions in cell viability were observed in mammalian breast epithelial cells (MCF10A) exposed to a dielectric barrier discharge (DBD) plasma [5,45]. The direct exposure of keratinocyte cells (HaCaT) to argon-generated cold atmospheric plasma resulted in a significant decrease in cell viability [46]. Likewise, fibroblast cells (3T3 cells) exposed to helium-generated cold atmospheric plasma showed a noticeable reduction in proliferation [47]. In the case of cell proliferation, the exposure of keratinocyte cells to helium-, oxygen-, and argon-generated cold atmospheric plasma led to reduced proliferation, increased intracellular and extracellular reactive species, and cell cycle arrest in the G2/M phase [48,49]. The decrease in cell proliferation resulted from the reactive species, ultraviolet and electromagnetic radiation, and heat released from plasma, as described in a previous study [1]. The air flow rates of 3 and 5 L/min were chosen for additional experiments as they had less than a 30% impact on cell viability and proliferation. In addition, it is important to maintain cell viability to accurately observe the effects of oxidative or nitrosative stress. Using nontoxic doses was chosen to monitor the impact on cells without compromising their overall health. In addition, nontoxic doses are more representative of physiological conditions. Using toxic doses may induce responses that are not reflective of typical cellular behavior. Moreover, previous reports indicated that the atmospheric nonthermal argon plasma at an air flow rate of 2–5 L/min utilized in a clinical study showed efficiency in inducing wound closure and reducing pruritus without external side effects [11,50,51,52].

Since the reactive species were predominately generated from plasma, further experiments were conducted to measure extracellular and intracellular ROS and RNS. We observed a significant induction of extracellular ROS and RNS as well as intracellular RNS after plasma exposure. It could be concluded that the plasma used in this study predominantly activated RNS production rather than ROS production in the murine fibroblast cells. This finding is consistent with the study by Thana et al., 2019 [21], where they investigated the chemical characterization of plasma from the same device in the current study. They found that H_2_O_2_ production was lower in the gas phase compared to the liquid phase, as long-lifetime plasma species, especially O_3_, interacted with water molecules to produce H_2_O_2_. Nitrogen species (nitrate and nitrite) were predominantly generated in direct proportion to the exposure time. Plasma released a controllable amount of NO, OH, O, and O_3_, and their interaction with water in the surrounding environment produced H_2_O_2_, NO_2_^–^, NO_3_^–^, HNO_2_, and HNO_3_. In a study by Shekhter et al. in 2005 [53], a predominant amount of nitric oxide was generated by air plasma, which contributed to wound healing in a mice model. On the other hand, when plasma was generated from helium or argon as a carrier gas, it produced inert gas, electrons, and atoms that reacted with the liquid phase in the environment, resulting in the generation of the most ROS [44]. The dissociation of water or liquid molecules, likely present in the culture medium, was activated by plasma treatment, leading to the formation of short-lived species lasting a few nanoseconds. These molecules then subsequently generated transient and more stable species with lifetimes longer than one second [54]. Considering that atmospheric air consists predominantly of nitrogen, followed by oxygen, it is probable that ROS may be generated in smaller amounts compared to RNS. Additionally, the reduced production of reactive species, especially ROS, might result from the activation of the host defense system. A study by Schmidt et al. in 2015 [55] revealed that shorter treatment times (20 s) of an argon plasma jet on keratinocyte cells resulted in significantly lower ROS formation levels due to the activation of the antioxidant process through the *Nrf2* signaling pathway. However, the host defense system against reactive species was not investigated in the current study, so further research is needed to study the antioxidant response after exposure to cells with plasma.

Intracellular RNS have been reported to interfere with cell proliferation through interactions with cyclin proteins, cyclin kinase activity, growth factors, and their receptors [56,57]. Additionally, damage caused by reactive species to genetic material can lead to various consequences, including mutations, chromosomal aberrations, and cell death, which may have serious implications for an organism’s health, including an increased risk of cancer and other diseases [58]. Fibroblast cells are susceptible to DNA damage and mutations due to ROS and RNS generated from both endogenous and exogenous sources. Therefore, we quantified DNA damage and 8-OHdG in plasma-exposed cells. The results demonstrated a significant induction of DNA damage in plasma-exposed cells at an intensity of 10 coupled with air flow rates of 3 and 5 L/min. This induction of DNA damage in murine fibroblast cells might be caused by extracellular and intracellular reactive species generated after plasma exposure to culture media and the cells. Consistent with the study by Steuer et al. in 2018 [59], the effect of plasma on the cell membrane could be mediated by reactive species in aqueous solutions, such as media or extracellular fluids. These short-lived reactive species can react with organic components like serum or amino acids to produce long-lived reactive species, most likely amino acid and protein hydroperoxides [59,60]. Furthermore, extracellular RNS such as nitric oxide are tiny and hydrophobic molecules that can traverse cellular membranes virtually unhindered [61]. Although several reactive species can directly attack genetic material, DNA strands are not highly reactive to RNS, particularly nitric oxide radicals. Instead, several RNS are formed by their reaction with intracellular oxygen radicals produced under oxidative phosphorylation in mitochondria, leading to the generation of potent DNA-damaging effects [62]. 

Regarding the 8-OHdG level, no significant production of 8-OHdG was observed in the plasma-exposed cells. Two events could explain this finding. First, the level of reactive species generated from plasma was not sufficient to induce this event. Second, oxidative DNA damage, such as 8-OHdG, is primarily generated by the attack of hydroxyl radicals on guanosine bases, but other reactive oxygen species (ONOO^−^, ^•^OOH, CO_3_^•−^ or ^1^O_2_) may also play an intermediate role and increase the rate of 8-OHdG formation [63]. The peroxynitrite anion (ONOO^−^), derived from the reaction between H_2_O_2_ and nitrite (NO_2_^−^) or superoxide (O_2_^−•^) with nitric oxide radical (NO), could be an intermediating molecule in inducing 8-OHdG [64]. A previous study by Kurita et al. in 2020 [65] showed a significant induction of 8-OHdG as well as DNA strand breaks after the irradiation of a human lung cancer cell line with cold plasma that used helium as a carrier gas. It is a crucial time point for understanding the dynamics of oxidative damage and repair processes in DNA. At 24 h, the accumulation of 8-OHdG may reach a level that is detectable and indicative of the extent of oxidative stress experienced by the cells or tissues. However, the time of detection of 8-OHdG in cells can vary depending on the experimental conditions and the sensitivity of the detection method used. Typically, after inducing oxidative stress in cells, 8-OHdG can be detected within a few hours to several days, depending on the extent of the damage and the repair mechanisms in the cells. According to the study by Kim et al., 2017 [66], the detection of 8-OHdG was performed at 24 h after the exposure of keratinocyte cells to nonthermal DBD plasma. The result showed a significant induction of 8-oxodG in the plasma treatment group. In addition, *OGG1* expression in keratinocyte cells exposed to DBD plasma was reduced compared with control cells. This might be indicative of the detection of 8-OHdG. Likewise, in the work by Kang et al., 2020 [67], keratinocyte cells were exposed to argon plasma; then, 8-OHdG was quantified at 24 h after plasma exposure, which is consistent with the amount of ROS production. From these supporting data, the limitation of our study is the lack of an experiment to confirm the effect of plasma on *OGG1*’s function. Therefore, we assumed that the nonsignificant induction of 8-OHdG on plasma-exposed cells probably resulted from 1) the low level of reactive species, especially ROS, to generate or adduct with the guanine base or 2) the activity of *OGG1* that removes the adduct base. 

Our study found a significant increase in DNA damage in a TUNEL assay and the fluorescent intensity of gamma H2AX (γ-H2AX), indicating double-strand breaks (DSBs), when the cells were exposed to plasma. There are two possibilities for this event. First, the composition of plasma such as reactive species could attack the DNA strand and induce the breaking of DNA. The other is the induction of cell stress that induces early apoptotic cell death, resulting in the induction of DNA fragmentation in cells. While TUNEL staining is commonly used as a method for detecting DNA damage (DNA fragmentation) and specifically for identifying apoptotic cells under appropriate conditions, it is important to note that TUNEL staining is not limited to apoptotic cell detection. TUNEL staining has been widely adopted as the method of choice for detecting apoptosis in situ. However, it can also be used to detect DNA damage associated with nonapoptotic events, such as necrotic cell death induced by exposure to toxic compounds and other insults [68]. Additionally, strand breaks detected by the TUNEL assay are sometimes not indicative of apoptosis but instead represent sites of temporary damage and potential repair [69]. A positive result in the TUNEL assay is not solely attributable to ROS but also to RNS and other mediators. Among the most prominent RNS is nitric oxide (NO). Although NO itself does not directly harm DNA, it can combine with superoxide (O_2_^−^) to generate peroxynitrite (ONOO^−^), an extremely reactive molecule capable of inducing DNA strand breaks, base modifications, and other forms of DNA damage. Peroxynitrite, in particular, is known to trigger DNA fragmentation by causing both single- and double-strand breaks in DNA strands. This molecule can directly interact with DNA, leading to the creation of DNA strand breaks and other forms of DNA damage [70]. The TUNEL assay identifies these DNA breaks by attaching a fluorescent marker to the free 3′-OH ends of fragmented DNA. Consequently, if RNS are present and responsible for DNA fragmentation, the TUNEL assay would yield a positive outcome.

The DNA damage induced by plasma generated from plasma in our study might be among the main mutagenic processes playing a role in the development of various diseases. Therefore, the mutagenicity of plasma was further evaluated. We found that all intensities of plasma under all air flow rates were not classified as mutagenic (mutagenic index less than 2); however, plasma exposure at an intensity of 10 pulses under an air flow rate of 5 L/min significantly elevated the revertant colonies of TA100. While plasma did not classify as a mutagen, there is still a possibility of it causing mutations depending on the amount and duration of exposure. The mutagenicity of cold atmospheric plasma by the Ames test has been explored to a lesser extent. In a study by Patenall et al. (2021) [30], a modified Ames test using *E. coli* strains WP2 was conducted. The results showed that mutations in the bacteria were significantly induced by exposing them to helium plasma for longer durations (10 min). However, this study used a different type of carrier gas compared to our study, which may result in distinct levels of chemical composition. Additionally, in our study, we opted to compare this novel plasma type with other established tests, albeit with the shared objective of mutation detection. Our findings are in line with previous studies indicating that cold atmospheric plasma (CAP) does not induce mutagenicity in V79 Chinese hamster cells assessed by the HPRT assay. However, it is important to note that comparisons between our results and those of the HPRT assay should consider the difference in the organisms used in these assays [71]. Although the bacterial strains TA102 and TA104 are more specific to oxidative damage, other components of plasma, such as UV photons, neutral or excited atoms and molecules, negative and positive ions, and free electrons, might affect the cells and cause DNA damage beyond oxidative damage. A previous study by Thana et al., 2019 [21], found that the CAPJ in the same device as our study was composed not only of reactive species but also UVB (280–315 nm) and UVA (315–400 nm). According to Waris and Ahsan’s 2006 review article [72], mutations resulting from oxidative DNA damage encompass various specifically oxidized purines and pyrimidines, alkali-labile sites, single-strand breaks, and instability caused either directly or through repair processes. Research indicates that although ROS modifies all four DNA bases, mutations typically involve changes in GC base pairs, with modifications in AT base pairs seldom leading to mutations. These mutations are predominantly base pair substitutions, with base deletions and insertions occurring less frequently. Hence, the mutagenicity testing in both TA98 and TA100 was initially performed for adequate bacterial mutagenicity screening. 

The genotoxic response caused by nonthermal plasma was studied with different types of plasma sources and treatment conditions. The exposure of plasmid DNA solutions to various helium plasma jets for durations ranging from 10 to 60 s resulted in a progressive increase in both single- and double-strand breaks, alongside a rise in the percentage of oxygen admixture. This observation indicates that the reactive species generated in the plasma plume can directly cause damage to the DNA material [73]. The mutation rate of a plasma-based mutagenesis tool was quantitatively assessed. This revealed that the plasma system which was based on helium gas caused significantly higher DNA damage and resulted in higher mutation rates in comparison to standard mutagens, such as ultraviolet radiation, 4-nitroquinoline-1-oxide (4-NQO), and N-methyl-N′-nitro-N-nitrosoguanidine (MNNG) [74]. Using atmospheric air as a carrier gas, similarly to our study, Blackert et al., 2013 [75], found that a plasma treatment time of one minute caused a significant increase in DNA damage in HaCaT cells compared to untreated control cells. When human esophageal cancer (KYSE-30) cells were exposed to the plasma jet device-generated cold atmospheric plasma for 60 s, a remarkably significant rise in DNA damage was observed in comparison to the untreated cells [76]. However, comparing our results with others proves challenging due to the variations in plasma sources, cell types, treatment conditions, and plasma intensity reported in the literature. Nevertheless, it appears that nonthermal plasma induces minimal genotoxicity in cells, as evidenced by existing studies.

## 5. Conclusions

In conclusion, the percentage of cell viability and cell proliferation was significantly decreased after treating NIH3T3 murine fibroblast cells with plasma generated from Nightingale. The predominant production of extracellular and intracellular reactive species was found. Although we observed DNA strand breaks, the plasma was not classified as a mutagen in the Ames test under the plasma intensity and exposure duration used in our study. It is essential to carefully select the appropriate plasma intensity for specific applications to minimize any adverse effects caused by the reactive species generated from plasma. However, this study has limitations and could be expanded for a more comprehensive outlook. Further investigation into mutagenicity could include strains such as TA102 or TA104, which are specific to oxidative damage. Moreover, experimental designs could be extended to animal models, as these encompass entire organisms with multiple interacting systems, unlike the simplified and controlled environment of cell culture models.

## Figures and Tables

**Table 1:** The number of revertant colonies showing in TA98 and TA100 strains from Ames’s test is expressed as mean and standard error of mean (SEM). Phosphate buffer was used as a negative control. 2-(2-furyl)-3-(5-nitro-2-furyl)-acrylamide (AF-2) dissolved in dimethyl sulfoxide (DMSO) was also used as a positive mutagen.

Treatment		His+ Revertant Colonies	
		Strain TA98	Strain TA100
Phosphate buffer		20.7 ± 4.3	88.6 ± 3.9
2-(2-furyl)-3-(5-nitro-2-furyl)-acrylamide (AF-2)		246.1 ± 44.4 ***	511.8 ± 53.3 ***
Air flow 3 L/min	Intensity of 0 pulses	18.6 ± 3.4	86.9 ± 0.1
	Intensity of 4 pulses	17.7 ± 2.5	80.8 ± 3.3
	Intensity of 7 pulses	21.2 ± 4.2	84.6 ± 9.6
	Intensity of 10 pulses	23.8 ± 5.1	94.7 ± 2.9
Air flow 5 L/min	Intensity of 0 pulses	21.8 ± 4.5	84.7 ± 5.7
	Intensity of 4 pulses	20.6 ± 3.9	91.4 ± 3.1
	Intensity of 7 pulses	24.0 ± 4.2	92.2 ± 4.1
	Intensity of 10 pulses	20.8 ± 4.5	104.7 ± 4.4 *^,#^

* and *** indicate a statistical difference, comparing the experimental group to the negative control (* *p* value < 0.05 and *** *p* value < 0.001). ^#^ indicates a statistical difference, comparing plasma intensities of 4, 7, and 10 pulses to a plasma intensity of 0 pulses at the same flow rate (^#^ *p* value < 0.05). Data are representative of three independent experiments.

## Data Availability

Data is contained within the article.

## References

[1] Haertel B., von Woedtke T., Weltmann K.-D., Lindequist U. (2014). Non-thermal atmospheric-pressure plasma possible application in wound healing. Biomol. Ther..

[2] Bernhardt T., Semmler M.L., Schäfer M., Bekeschus S., Emmert S., Boeckmann L. (2019). Plasma Medicine: Applications of Cold Atmospheric Pressure Plasma in Dermatology. Oxidative Med. Cell. Longev..

[3] Gan L., Zhang S., Poorun D., Liu D., Lu X., He M., Duan X., Chen H. (2018). Medical applications of nonthermal atmospheric pressure plasma in dermatology. J. Dtsch. Dermatol. Ges..

[4] Ziuzina D., Los A., Bourke P., Fetsch A. (2018). Chapter 12—Inactivation of Staphylococcus aureus in Foods by Thermal and Nonthermal Control Strategies. Staphylococcus aureus.

[5] Kalghatgi S., Kelly C.M., Cerchar E., Torabi B., Alekseev O., Fridman A., Friedman G., Azizkhan-Clifford J. (2011). Effects of non-thermal plasma on mammalian cells. PLoS ONE.

[6] Ahn H.J., Kim K.I., Hoan N.N., Kim C.H., Moon E., Choi K.S., Yang S.S., Lee J.S. (2014). Targeting cancer cells with reactive oxygen and nitrogen species generated by atmospheric-pressure air plasma. PLoS ONE.

[7] Joh H.M., Kim S.J., Chung T.H., Leem S.H. (2013). Comparison of the characteristics of atmospheric pressure plasma jets using different working gases and applications to plasma-cancer cell interactions. AIP Adv..

[8] Lou B.-S., Hsieh J.-H., Chen C.-M., Hou C.-W., Wu H.-Y., Chou P.-Y., Lai C.-H., Lee J.-W. (2020). Helium/Argon-Generated Cold Atmospheric Plasma Facilitates Cutaneous Wound Healing. Front. Bioeng. Biotechnol..

[9] Kang S.U., Choi J.W., Chang J.W., Kim K.i., Kim Y.S., Park J.K., Kim Y.E., Lee Y.S., Yang S.S., Kim C.-H. (2017). N2 non-thermal atmospheric pressure plasma promotes wound healing in vitro and in vivo: Potential modulation of adhesion molecules and matrix metalloproteinase-9. Exp. Dermatol..

[10] Shi X.M., Xu G.M., Zhang G.J., Liu J.R., Wu Y.M., Gao L.G., Yang Y., Chang Z.S., Yao C.W. (2018). Low-temperature Plasma Promotes Fibroblast Proliferation in Wound Healing by ROS-activated NF-κB Signaling Pathway. Curr. Med. Sci..

[11] Isbary G., Morfill G., Schmidt H.U., Georgi M., Ramrath K., Heinlin J., Karrer S., Landthaler M., Shimizu T., Steffes B. (2010). A first prospective randomized controlled trial to decrease bacterial load using cold atmospheric argon plasma on chronic wounds in patients. Br. J. Dermatol..

[12] Isbary G., Stolz W., Shimizu T., Monetti R., Bunk W., Schmidt H.U., Morfill G.E., Klämpfl T.G., Steffes B., Thomas H.M. (2013). Cold atmospheric argon plasma treatment may accelerate wound healing in chronic wounds: Results of an open retrospective randomized controlled study in vivo. Clin. Plasma Med..

[13] Laroussi M., Lu X., Keidar M. (2017). Perspective: The physics, diagnostics, and applications of atmospheric pressure low temperature plasma sources used in plasma medicine. J. Appl. Phys..

[14] Lietz A.M., Kushner M.J. (2018). Molecular admixtures and impurities in atmospheric pressure plasma jets. J. Appl. Phys..

[15] Di Meo S., Reed T.T., Venditti P., Victor V.M. (2016). Harmful and Beneficial Role of ROS. Oxidative Med. Cell. Longev..

[16] Pham-Huy L.A., He H., Pham-Huy C. (2008). Free radicals, antioxidants in disease and health. Int. J. Biomed. Sci..

[17] Lobo V., Patil A., Phatak A., Chandra N. (2010). Free radicals, antioxidants and functional foods: Impact on human health. Pharmacogn. Rev..

[18] Braný D., Dvorská D., Halašová E., Škovierová H. (2020). Cold Atmospheric Plasma: A Powerful Tool for Modern Medicine. Int. J. Mol. Sci..

[19] Boehm D., Bourke P. (2019). Safety implications of plasma-induced effects in living cells—A review of in vitro and in vivo findings. Biol. Chem..

[20] Thana P., Kuensaen C., Poramapijitwat P., Sarapirom S., Yu L., Boonyawan D. (2020). A compact pulse-modulation air plasma jet for the inactivation of chronic wound bacteria: Bactericidal effects & host safety. Surf. Coat. Technol..

[21] Thana P., Wijaikhum A., Poramapijitwat P., Kuensaen C., Meerak J., Ngamjarurojana A., Sarapirom S., Boonyawan D. (2019). A compact pulse-modulation cold air plasma jet for the inactivation of chronic wound bacteria: Development and characterization. Heliyon.

[22] Herting S.M., Monroe M.B.B., Weems A.C., Briggs S.T., Fletcher G.K., Blair S.E., Hatch C.J., Maitland D.J. (2021). In vitro cytocompatibility testing of oxidative degradation products. J. Bioact. Compat. Polym..

[23] (2009). Biological Evaluation of Medical Devices-Part 5: Tests for In Vitro Cytotoxicity.

[24] Guerrero-Preston R., Ogawa T., Uemura M., Shumulinsky G., Valle B.L., Pirini F., Ravi R., Sidransky D., Keidar M., Trink B. (2014). Cold atmospheric plasma treatment selectively targets head and neck squamous cell carcinoma cells. Int. J. Mol. Med..

[25] Franken N.A.P., Rodermond H.M., Stap J., Haveman J., van Bree C. (2006). Clonogenic assay of cells in vitro. Nat. Protoc..

[26] Vargas-Maya N.I., Padilla-Vaca F., Romero-González O.E., Rosales-Castillo E.A.S., Rangel-Serrano Á., Arias-Negrete S., Franco B. (2021). Refinement of the Griess method for measuring nitrite in biological samples. J. Microbiol. Methods.

[27] Howland J.L. (1997). Methods in nitric oxide research. Biochem. Educ..

[28] Jang T.W., Choi J.S., Park J.H. (2020). Protective and inhibitory effects of acteoside from Abeliophyllum distichum Nakai against oxidative DNA damage. Mol. Med. Rep..

[29] OECD (2020). Test No. 471: Bacterial Reverse Mutation Test.

[30] Patenall B.L., Hathaway H.J., Laabei M., Young A.E., Thet N.T., Jenkins A.T.A., Short R.D., Allinson S.L. (2021). Assessment of mutations induced by cold atmospheric plasma jet treatment relative to known mutagens in *Escherichia coli*. Mutagenesis.

[31] Mortelmans K., Zeiger E. (2000). The Ames Salmonella/microsome mutagenicity assay. Mutat. Res..

[32] Santos F.V., Colus I.M., Silva M.A., Vilegas W., Varanda E.A. (2006). Assessment of DNA damage by extracts and fractions of Strychnos pseudoquina, a Brazilian medicinal plant with antiulcerogenic activity. Food Chem. Toxicol..

[33] Zarkovic N. (2020). Roles and Functions of ROS and RNS in Cellular Physiology and Pathology. Cells.

[34] Di Meo S., Reed T.T., Venditti P., Victor V.M. (2016). Role of ROS and RNS Sources in Physiological and Pathological Conditions. Oxidative Med. Cell. Longev..

[35] (2003). Biological Evaluation of Medical Devices-Part 3: Tests for Genotoxicity, Carcinogenicity and Reproductive Toxicity.

[36] Carter M., Shieh J., Carter M., Shieh J. (2015). Chapter 14—Cell Culture Techniques. Guide to Research Techniques in Neuroscience.

[37] Laroussi M. (2019). Effects of PAM on select normal and cancerous epithelial cells. Plasma Res. Express.

[38] Lin A., Gorbanev Y., De Backer J., Van Loenhout J., Van Boxem W., Lemière F., Cos P., Dewilde S., Smits E., Bogaerts A. (2019). Non-Thermal Plasma as a Unique Delivery System of Short-Lived Reactive Oxygen and Nitrogen Species for Immunogenic Cell Death in Melanoma Cells. Adv. Sci..

[39] Brehmer F., Haenssle H.A., Daeschlein G., Ahmed R., Pfeiffer S., Görlitz A., Simon D., Schön M.P., Wandke D., Emmert S. (2015). Alleviation of chronic venous leg ulcers with a hand-held dielectric barrier discharge plasma generator (PlasmaDerm^®^ VU-2010): Results of a monocentric, two-armed, open, prospective, randomized and controlled trial (NCT01415622). J. Eur. Acad. Dermatol. Venereol..

[40] Xu G.M., Shi X.M., Cai J.F., Chen S.L., Li P., Yao C.W., Chang Z.S., Zhang G.J. (2015). Dual effects of atmospheric pressure plasma jet on skin wound healing of mice. Wound Repair. Regen..

[41] Stratmann B., Costea T.C., Nolte C., Hiller J., Schmidt J., Reindel J., Masur K., Motz W., Timm J., Kerner W. (2020). Effect of Cold Atmospheric Plasma Therapy vs Standard Therapy Placebo on Wound Healing in Patients With Diabetic Foot Ulcers: A Randomized Clinical Trial. JAMA Netw. Open.

[42] Kluge S., Bekeschus S., Bender C., Benkhai H., Sckell A., Below H., Stope M.B., Kramer A. (2016). Investigating the Mutagenicity of a Cold Argon-Plasma Jet in an HET-MN Model. PLoS ONE.

[43] Wende K., Bekeschus S., Schmidt A., Jatsch L., Hasse S., Weltmann K.D., Masur K., von Woedtke T. (2016). Risk assessment of a cold argon plasma jet in respect to its mutagenicity. Mutat. Res. Genet. Toxicol. Environ. Mutagen..

[44] Takamatsu T., Uehara K., Sasaki Y., Miyahara H., Matsumura Y., Iwasawa A., Ito N., Azuma T., Kohno M., Okino A. (2014). Investigation of reactive species using various gas plasmas. RSC Adv..

[45] Dobrynin D., Fridman G., Friedman G., Fridman A. (2009). Physical and biological mechanisms of direct plasma interaction with living tissue. New J. Phys..

[46] Straßenburg S., Greim U., Bussiahn R., Haertel B., Wende K., von Woedtke T., Lindequist U. (2013). Comparison of Biological Effects on Human Keratinocytes Using Different Plasma Treatment Regimes. Plasma Med..

[47] Lunov O., Zablotskii V., Churpita O., Chánová E., Syková E., Dejneka A., Kubinová Š. (2014). Cell death induced by ozone and various non-thermal plasmas: Therapeutic perspectives and limitations. Sci. Rep..

[48] Kim K.C., Piao M.J., Madduma Hewage S.R., Han X., Kang K.A., Jo J.O., Mok Y.S., Shin J.H., Park Y., Yoo S.J. (2016). Non-thermal dielectric-barrier discharge plasma damages human keratinocytes by inducing oxidative stress. Int. J. Mol. Med..

[49] Wende K., Straßenburg S., Haertel B., Harms M., Holtz S., Barton A., Masur K., von Woedtke T., Lindequist U. (2014). Atmospheric pressure plasma jet treatment evokes transient oxidative stress in HaCaT keratinocytes and influences cell physiology. Cell Biol. Int..

[50] Heinlin J., Isbary G., Stolz W., Zeman F., Landthaler M., Morfill G., Shimizu T., Zimmermann J.L., Karrer S. (2013). A randomized two-sided placebo-controlled study on the efficacy and safety of atmospheric non-thermal argon plasma for pruritus. J. Eur. Acad. Dermatol. Venereol..

[51] Ulrich C., Kluschke F., Patzelt A., Vandersee S., Czaika V.A., Richter H., Bob A., Hutten J., Painsi C., Hüge R. (2015). Clinical use of cold atmospheric pressure argon plasma in chronic leg ulcers: A pilot study. J. Wound Care.

[52] Heinlin J., Zimmermann J.L., Zeman F., Bunk W., Isbary G., Landthaler M., Maisch T., Monetti R., Morfill G., Shimizu T. (2013). Randomized placebo-controlled human pilot study of cold atmospheric argon plasma on skin graft donor sites. Wound Repair. Regen..

[53] Shekhter A.B., Serezhenkov V.A., Rudenko T.G., Pekshev A.V., Vanin A.F. (2005). Beneficial effect of gaseous nitric oxide on the healing of skin wounds. Nitric Oxide.

[54] Buxton G.V., Greenstock C.L., Helman W.P., Ross A.B. (1988). Critical Review of rate constants for reactions of hydrated electrons, hydrogen atoms and hydroxyl radicals (OH/O−) in Aqueous Solution. J. Phys. Chem. Ref. Data.

[55] Schmidt A., Dietrich S., Steuer A., Weltmann K.D., von Woedtke T., Masur K., Wende K. (2015). Non-thermal plasma activates human keratinocytes by stimulation of antioxidant and phase II pathways. J. Biol. Chem..

[56] Martinez M.C., Andriantsitohaina R. (2009). Reactive Nitrogen Species: Molecular Mechanisms and Potential Significance in Health and Disease. Antioxid. Redox Signal..

[57] Milsom A.B., Jones C.J., Goodfellow J., Frenneaux M.P., Peters J.R., James P.E. (2002). Abnormal metabolic fate of nitric oxide in Type I diabetes mellitus. Diabetologia.

[58] Phaniendra A., Jestadi D.B., Periyasamy L. (2015). Free radicals: Properties, sources, targets, and their implication in various diseases. Indian J. Clin. Biochem..

[59] Steuer A., Wolff C.M., von Woedtke T., Weltmann K.-D., Kolb J.F. (2018). Cell stimulation versus cell death induced by sequential treatments with pulsed electric fields and cold atmospheric pressure plasma. PLoS ONE.

[60] Gracanin M., Lam M.A., Morgan P.E., Rodgers K.J., Hawkins C.L., Davies M.J. (2011). Amino acid, peptide, and protein hydroperoxides and their decomposition products modify the activity of the 26S proteasome. Free Radic. Biol. Med..

[61] Möller M.N., Cuevasanta E., Orrico F., Lopez A.C., Thomson L., Denicola A. (2019). Diffusion and Transport of Reactive Species Across Cell Membranes. Adv. Exp. Med. Biol..

[62] Sawa T., Ohshima H. (2006). Nitrative DNA damage in inflammation and its possible role in carcinogenesis. Nitric Oxide.

[63] Giorgio M., Dellino G.I., Gambino V., Roda N., Pelicci P.G. (2020). On the epigenetic role of guanosine oxidation. Redox Biol..

[64] Jena N.R., Mishra P.C. (2007). Formation of 8-nitroguanine and 8-oxoguanine due to reactions of peroxynitrite with guanine. J. Comput. Chem..

[65] Kurita H., Haruta N., Uchihashi Y., Seto T., Takashima K. (2020). Strand breaks and chemical modification of intracellular DNA induced by cold atmospheric pressure plasma irradiation. PLoS ONE.

[66] Kim K.C., Ruwan Kumara M.H.S., Kang K.A., Piao M.J., Oh M.C., Ryu Y.S., Jo J.O., Mok Y.S., Shin J.H., Park Y. (2017). Exposure of keratinocytes to non-thermal dielectric barrier discharge plasma increases the level of 8-oxoguanine via inhibition of its repair enzyme. Mol. Med. Rep..

[67] Kang K.A., Piao M.J., Eom S., Yoon S.-Y., Ryu S., Kim S.B., Yi J.M., Hyun J.W. (2020). Non-thermal dielectric-barrier discharge plasma induces reactive oxygen species by epigenetically modifying the expression of NADPH oxidase family genes in keratinocytes. Redox Biol..

[68] Loo D.T., Didenko V.V. (2002). TUNEL Assay. In Situ Detection of DNA Damage. Methods in Molecular Biology.

[69] Hornsby P.J., Didenko V.V. (2011). In situ ligation: A decade and a half of experience. Methods Mol. Biol..

[70] Burney S., Caulfield J.L., Niles J.C., Wishnok J.S., Tannenbaum S.R. (1999). The chemistry of DNA damage from nitric oxide and peroxynitrite. Mutat. Res..

[71] Boxhammer V., Li Y.F., Köritzer J., Shimizu T., Maisch T., Thomas H.M., Schlegel J., Morfill G.E., Zimmermann J.L. (2013). Investigation of the mutagenic potential of cold atmospheric plasma at bactericidal dosages. Mutat. Res. Genet. Toxicol. Environ. Mutagen..

[72] Waris G., Ahsan H. (2006). Reactive oxygen species: Role in the development of cancer and various chronic conditions. J. Carcinog..

[73] Alkawareek M.Y., Alshraiedeh N.a.H., Higginbotham S., Flynn P.B., Algwari Q.T., Gorman S.P., Graham W.G., Gilmore B.F. (2014). Plasmid DNA Damage Following Exposure to Atmospheric Pressure Nonthermal Plasma: Kinetics and Influence of Oxygen Admixture. Plasma Med..

[74] Zhang X., Zhang C., Zhou Q.Q., Zhang X.F., Wang L.Y., Chang H.B., Li H.P., Oda Y., Xing X.H. (2015). Quantitative evaluation of DNA damage and mutation rate by atmospheric and room-temperature plasma (ARTP) and conventional mutagenesis. Appl. Microbiol. Biotechnol..

[75] Blackert S., Haertel B., Wende K., von Woedtke T., Lindequist U. (2013). Influence of non-thermal atmospheric pressure plasma on cellular structures and processes in human keratinocytes (HaCaT). J. Dermatol. Sci..

[76] Estarabadi H., Atyabi S.A., Tavakkoli S., Noormohammadi Z., Gholami M.R., Ghiaseddin A., Irani S. (2021). Cold atmospheric plasma induced genotoxicity and cytotoxicity in esophageal cancer cells. Mol. Biol. Rep..

